# Role of CaMKII and PKA in Early Afterdepolarization of Human Ventricular Myocardium Cell: A Computational Model Study

**DOI:** 10.1155/2016/4576313

**Published:** 2016-12-08

**Authors:** Ling Dai, Yunliang Zang, Dingchang Zheng, Ling Xia, Yinglan Gong

**Affiliations:** ^1^Department of Biomedical Engineering, Zhejiang University, Hangzhou, China; ^2^Health and Wellbeing Academy, Faculty of Medical Science, Anglia Ruskin University, Chelmsford CM1 1SQ, UK

## Abstract

Early afterdepolarization (EAD) plays an important role in arrhythmogenesis. Many experimental studies have reported that Ca^2+^/calmodulin-dependent protein kinase II (CaMKII) and *β*-adrenergic signaling pathway are two important regulators. In this study, we developed a modified computational model of human ventricular myocyte to investigate the combined role of CaMKII and *β*-adrenergic signaling pathway on the occurrence of EADs. Our simulation results showed that (1) CaMKII overexpression facilitates EADs through the prolongation of late sodium current's (*I*_NaL_) deactivation progress; (2) the combined effect of CaMKII overexpression and activation of *β*-adrenergic signaling pathway further increases the risk of EADs, where EADs could occur at shorter cycle length (2000 ms versus 4000 ms) and lower rapid delayed rectifier K^+^ current (*I*_Kr_) blockage (77% versus 85%). In summary, this study computationally demonstrated the combined role of CaMKII and *β*-adrenergic signaling pathway on the occurrence of EADs, which could be useful for searching for therapy strategies to treat EADs related arrhythmogenesis.

## 1. Introduction

Early afterdepolarizations (EADs) are triggered before the completion of repolarization [1] and associated with polymorphic ventricular tachyarrhythmia for long QT syndrome patients [2]. Prolongation of action potential duration (APD) and recovery of L-type Ca^2+^ current have been reported as two important factors for the occurrence of EADs [3]. It is also known that the increase of inward currents (e.g., *I*_CaL_ and late sodium current, *I*_NaL_) or the decrease of outward currents (e.g., rapid delayed rectifier K^+^ current, *I*_Kr_ and slow delayed rectifier K^+^ current, *I*_Ks_) at plateau membrane voltage could increase the probability of EADs events. Therefore, any factors that could change the intensity or time sequence of these currents may lead to the occurrence of EADs [4–8].

Ca^2+^/calmodulin-dependent protein kinase II (CaMKII) is a key kinase in tuning cardiac excitation-contraction coupling. Its substrates include ion channels, transporters, and accessory proteins [9]. It has been reported that CaMKII phosphorylates *I*_CaL_, leading to increased amplitude and APD prolongation and facilitating the occurrence of EADs [10, 11]. CaMKII can also alter *I*_NaL_, transient outward K current (*I*_to_), SR Ca^2+^-ATPase (SERCA) [12], and ryanodine receptor (RyR) channels [10]. It would therefore be useful to understand and quantify these regulatory roles. However, it is very difficult for the laboratory experiments to achieve this. Computer modelling approaches provide alternative ways, allowing us to distinguish the most effective phosphorylation target of arrhythmogenesis, which would ultimately provide useful tool in searching for antiarrhythmia therapy.

It has been known that *β*-adrenergic signaling pathway regulates Ca^2+^ cycling partly via phosphorylation of *I*_CaL_ and phospholamban (PLB) [13]. *I*_CaL_ elevates intracellular Ca^2+^ ([Ca]_i_) and increases spontaneous Ca^2+^ release via the SERCA inhibition by PLB. The broken balance of Ca^2+^ cycling may contribute to the occurrence of EADs [14–16]. Volders et al. investigated the ionic mechanisms of *β*-adrenergic on the occurrence of EADs in canine ventricular myocyte and concluded that cellular Ca^2+^ overload and spontaneous SR Ca^2+^ release played important roles in EADs [17]. On the contrary, other studies reported that *β*-adrenergic agonists activate protein kinase A (PKA), which phosphorylates *I*_CaL_, RyR, PLB, SERCA, and *I*_Ks_, resulting in delayed afterdepolarization (DADs) [18]. These numerous targets and different temporal characteristics of phosphorylation effects complicate the mechanism analysis of *β*-adrenergic agonists in relation to EADs. Recently, different computational models have been used to investigate these complex interactions. Xie et al. developed a biophysically detailed rabbit model and found that the faster time course of *I*_CaL_ versus *I*_Ks_ increased ISO-induced transient EADs [19] and emphasized the importance of understanding the nonsteady state of kinetics in meditating *β*-adrenergic-induced EADs and arrhythmia. However, other targets including *I*_NaL_ have not been investigated thoroughly in their model. It is noted that, although the computational studies of EAD mechanisms have been widely taken, the majority of these published modelling studies have been developed based on nonhuman myocyte. Furthermore, to the best of our knowledge, there is no modelling study considering the combined effect of CaMKII overexpression and *β*-adrenergic agonists on EADs.

This study aimed to develop a modified computational model of human ventricular myocyte that integrates CaMKII and *β*-adrenergic signaling networks into a modified ORd's dynamic model [20], with which the combined role of CaMKII and *β*-adrenergic signaling pathway on the occurrence of EADs would be investigated.

## 2. Methods

### 2.1. Integration of CaMKII

Since there is a lack of experimental measurements of human ventricle CaMKII pathway, O'Hara et al. used the Hund-Decker-Rudy's dog model to describe CaMKII kinetics [21, 22]. Our model was developed from the O'Hara-Rudy dynamic model (ORd model) to integrate CaMKII pathway [20]. The following equations describe the CaMKII kinetic of human ventricle:

(1) CaMKbound=CaMK0·1−CaMKtrap1+KmCaM/Ca2+ss,CaMKactive=CaMKbound+CaMKtrap,dCaMKtrapdt=αCaMK·CaMKbound·CaMKbound+CaMKtrap−βCaMK·CaMKtrap,αCaMK=0.05 ms−1,  βCaMK=0.00068  ms−1,  CaMK0=0.05,  KmCaM=0.0015 mM.

The fraction of active CaMKII binding sites at equilibrium state (CaMK_0_) was set to 0.05 at the control state. CaMK_0_ of 0.12 was used to simulate CaMKII overexpression according to the study from Kohlhaas et al. [23].

### 2.2. Integration of *β*-Adrenergic Signaling Networks

The detailed description of *β*-adrenergic signaling networks could be found from the published study by Soltis and Saucerman [24]. PKA has been reported to phosphorylate *I*_CaL_, PLB, troponin *I*, RyR, myosin binding protein-C, protein phosphates Inhibitor-*I* [25], and *I*_Ks_ [24]. *I*_CaL_, PLB, and *I*_Ks_ were the three key factors in this study to model the inotropic effect of *β*-adrenergic related to potential EADs occurrence. Although the PKA phosphorylation of Na^+^/K^+^ ATPase current (*I*_NaK_) has been previously described in the computational models [19, 26], its effect was not included in this study partially because there is a lack of direct measurements of *I*_NaK_ for the normal human ventricle [20]. The phosphorylation by PKA to the three targets (*I*_CaL_, PLB, and *I*_Ks_) is described as follows:

(2) favail=0.017·LCCbPKAPfracLCCbp0+0.983,

(3) dss=11.0+exp⁡−V+vshift/4.230,

(4) ICaL=ICaL¯·d·1−ϕICaL,CaMK·f·1−n+fCa·n·jCa+ICaL,CaMK¯·d·ϕICaL,CaMK·fCaMK·1−n+fCa,CaMK·n·jCa·favail,

(5) ΦICaL,CaMK=11+Km,CaMK/CaMKactive,

(6) td=1.2·0.6+1exp⁡−0.05·V+6.0+exp⁡0.09·V+14.0.

Equation (2) shows the coefficient that represents the effect of PKA on *I*_CaL_ phosphorylation. In (3), the value of “*v*shift” was increased from 3.94 (no ISO application) to 10.0 (for saturated ISO application), which means that the steady state activation curve of *I*_CaL_ was moved left by 6.06 mV. The permeability of ion Ca^2+^ was increased by 10% with saturated ISO application. Equation (4) describes the augmentation of *I*_CaL_ amplitude by multiplying “*f*_avail_” with the value without PKA phosphorylation, and this represents how PKA regulates *I*_CaL_. In (6), the time constant of activation gate (*td*) was extended by 20% with saturated ISO application

(7) fracIKsavail=0.49·IKsPKAPfracIKsp0+0.51,

(8) GKs=fracIKsavail·GKs,

(9) Xs05=11.60·fracIKsavail,

(10) XS1,∞=11+exp⁡−V+Xs05/8.932.

Equation (7) describes the factor of phosphorylation to *I*_Ks_ by PKA, which was used to alter maximum conductance of *I*_Ks_ (GKs) in (8). Meanwhile, *I*_Ks_ state steady activation curve was adjusted by time dependent gate value through the factor “frac*I*_Ksavail_” in (9) and (10).

### 2.3. Combination of CaMKII and *β*-Adrenergic Signaling Networks

(11) fPKAPLB=PLB_PKAnfracPKAPLB0·14+34,

(12) fJupp=1.01.0+Km,CaMK·fPKAPLB/CaMKactive,

(13) Jup=1.0−fJupp·Jupnp+fJupp·Jupp−Jleak.

CaMK_active_ that is affected by CaMK_0_ as shown in (1) would influence *I*_CaL_ in (4) via the fraction of *I*_CaL_ channels phosphorylated by CaMKII (Φ_*I*_CaL,CaMK__). The fraction of SERCAs phosphorylated by CaMKII in (12) was affected by “fPKA_PLB_” and “CaMK_active_”, representing the effects of PKA and CaMKII on SERCAs, respectively. As shown in (13), SERCAs were separated into nonphosphorylated populations and CaMKII phosphorylated populations. Therefore, the total Ca^2+^ uptake via SERCAs was adjusted by these two networks simultaneously.

### 2.4. Simulation Strategy

In order to determine the potential targets in CaMKII-induced EADs, CaMKII was solely overexpressed by assigning the CaMK_0 _of 0.12 to specific targets (including *I*_NaL_, *I*_CaL_, *I*_CaK_, *I*_NaCa_, and *I*_to_), respectively, while *β*-adrenergic signaling was maintained inactive. Next, CaMKII overexpression was applied with ISO administration. 1 *μ*M ISO was applied in this study to simulate its effect on myocyte action potential and ion currents.

The cycle length of 2000 ms was used in this study since it was closer to normal human beat rhythm than the length of 4000 ms used in the experiments from Guo et al. [27]. In Guo et al.'s work, EADs appeared with *I*_Kr_ blockage of about 85% [27], which was used for comparison in this study. 500 cycles were performed when the simulation reached steady state. In each individual cycle length, the upper bound of solver step size was set 2 ms.

## 3. Results

### 3.1. Effect of CaMKII Overexpression on Ion Currents

#### 3.1.1. Late Sodium Current (*I*_NaL_)

**Figure 1:**
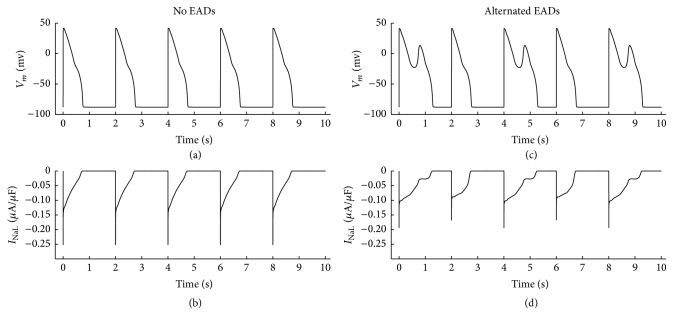
(a) No EAD was produced when CaMKII phosphorylation level was in control (CaMK_0_ = 0.05). (b) Corresponding *I*_NaL_ when no EADs occurred in (a). (c) Alternated EADs were produced when *I*_NaL_ phosphorylation by CaMKII was enhanced with CaMK_0_ of 0.12 and other targets' phosphorylation levels were in control (CaMK_0_ = 0.05). (d) Corresponding *I*_NaL_ when alternated EADs occurred in (c). Under these conditions, CL was 2000 ms and *I*_Kr_ was blocked by 85%.

As shown in Table 1 and Figure 1, with the overexpressed CaMK_0_ value to *I*_NaL_ of 0.12 and the normal CaMK_0_ value of 0.05 to other targets, the alternated EADs occur from our simulation with the cycle length (CL) of 2000 ms and *I*_Kr_ blockage of 85%.

As shown in Figure 1, I_NaL_ amplitudes alternated with overexpressed CaMK_0_. In beats with EADs, *I*_NaL_ amplitude was about 24% smaller (0.19 *μ*A/*μ*F versus 0.25 *μ*A/*μ*F) than these with normal CaMK_0_ value to *I*_NaL_ and in beats without EADs, *I*_NaL_ amplitude was also reduced by 32% (0.17 *μ*A/*μ*F versus 0.25 *μ*A/*μ*F). In Figure 1(d), when EADs occurred, *I*_NaL_ deactivated, which was about 168% of normal beats (1297 ms versus 773 ms) and about 170% of the control situation in Figure 1(b) (1297 ms versus 761 ms), indicating that overexpressed CaMKII phosphorylation level of *I*_NaL_ reduces its amplitude and prolongs *I*_NaL_ deactivation process. Furthermore, the results also indicate that the delayed deactivation of *I*_NaL_, rather than its amplitude variation, contributes to the formation of EADs.

#### 3.1.2. L-Type Calcium Current (*I*_CaL_)

Similarly, as shown in Table 1, with the overexpressed CaMK_0_ to *I*_CaL_ of 0.12 and the normal CaMK_0_ value of 0.05 to other targets, no EADs occur at CL of 2000 ms and *I*_Kr_ blockage of 85%, suggesting that the probability of EADs might have little relation with amplitude of *I*_CaL_. With the enhanced CaMK_0_ value to *I*_CaL_, the amplitudes of *I*_CaL_ only increased by 4.2% (1.74 *μ*A/*μ*F versus 1.67 *μ*A/*μ*F).

#### 3.1.3. K^+^ Current through the L-Type Ca^2+^ Channel (*I*_CaK_), Na^+^ Current through the L-Type Ca^2+^ Channel (*I*_CaNa_), and Transient Outward K^+^ Current (*I*_to_)

As above, CL was set to 2000 ms and *I*_Kr_ was blocked by 85%. As shown in Table 1, with enhanced CaMKII phosphorylation level (CaMK_0_ = 0.12) to different targets, *I*_CaK_ increased only by 6.5% (0.66 *μ*A/*μ*F versus 0.62 *μ*A/*μ*F), *I*_CaNa_ by 8.1% (0.40 *μ*A/*μ*F versus 0.37 *μ*A/*μ*F), and *I*_to_ by 3.2% (0.98 *μ*A/*μ*F versus 0.95 *μ*A/*μ*F). No EADs occurred under all these conditions.

### 3.2. Combined Effect of CaMKII Overexpression and *β*-Adrenergic Agonist

#### 3.2.1. Normal CaMKII and 1 *μ*M ISO

**Figure 2:**
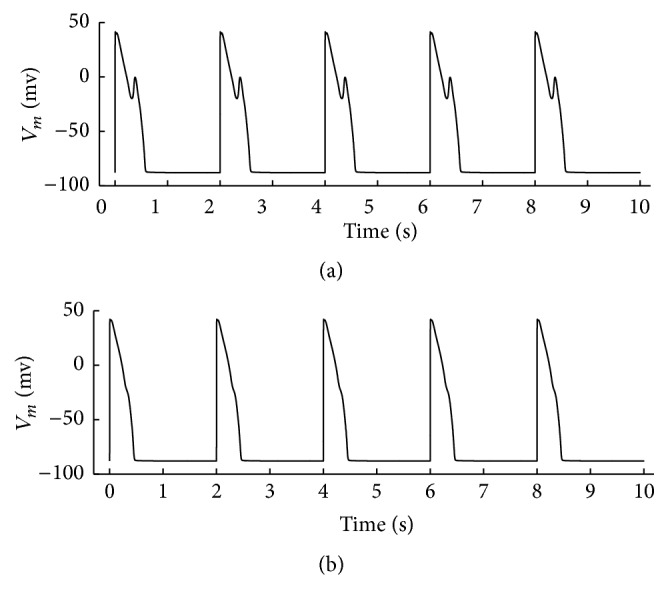
(a) EADs were induced when 1 *μ*M ISO was applied and *I*_Kr_ was blocked by 85%. In (b), EADs disappeared when *I*_Kr_ blockage was reduced to 77%. Cycle length was set 2000 ms and CaMKII phosphorylation level to all targets was kept control (CaMK_0_ = 0.05).

The action potentials with different *I*_Kr_ blockage level are shown in Figure 2. A fixed CL =2000 ms was used and CaMKII phosphorylation level to all targets was kept control (CaMK_0_ = 0.05). In Figure 2(a), with *I*_Kr_ blocked by 85%, stable EADs occurred with the application of ISO, indicating that *β*-adrenergic agonist facilitates EADs. The *I*_Kr_ blockage level decreased gradually from 85%, and, when it was decreased to 77%, as shown in Figure 2(b), these EADs disappeared, indicating that 77% was the threshold value for EADs disappearance in this setting. Therefore, 77% was used in following simulation to compare with previously published level of 85% [20]. These results are listed from row 2 to row 3 in Table 2.

#### 3.2.2. Enhanced CaMKII to *I*_NaL_ and 1 *μ*M ISO

**Figure 3:**
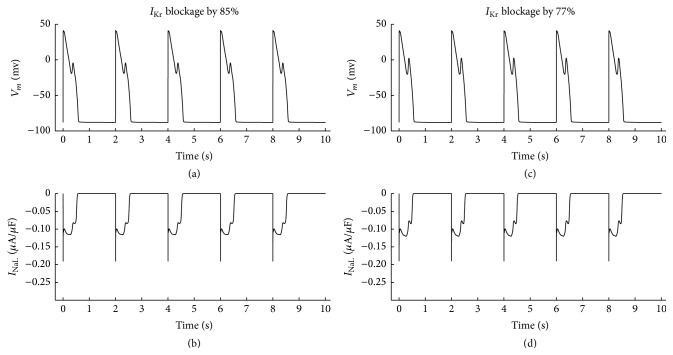
EADs occurred when *I*_Kr_ was blocked by 85% (a) and 77% (c); corresponding *I*_NaL_ when *I*_Kr_ was blocked by 85% (b) and 77% (d). CL was set 2000 ms, 1 *μ*M ISO was applied and CaMK_0_ for *I*_NaL_ was set 0.12 independently.

As shown in Figure 3, with enhanced CaMKII to *I*_NaL_ and 1 *μ*M ISO, when *I*_Kr_ was blocked by 85%, EADs were induced. The EADs were still observed when *I*_Kr_ was blocked by 77%, suggesting that *I*_NaL_ phosphorylation by CaMKII and ISO application together increased the probability of EADs. These results are listed from row 4 to row 5 in Table 2.

#### 3.2.3. Enhanced CaMKII to *I*_CaL_ and 1 *μ*M ISO

**Figure 4:**
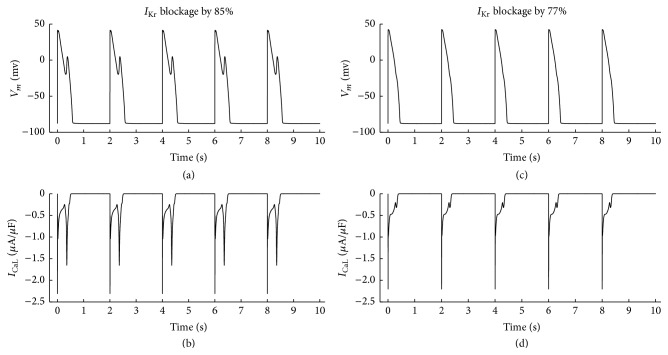
1 *μ*M ISO was applied and cycle length was 2000 ms. CaMK_0_ for *I*_CaL_ was 0.12 but CaMK_0_ for other targets was 0.05. EADs occurred when *I*_Kr_ was blocked by 85% in (a), but when *I*_Kr_ blockage was reduced to 77%, EADs vanished in (c). (b) *I*_CaL_ figures when EADs existed. (d) *I*_CaL_ figures when EADs vanished.

As shown in Figure 4, with enhanced CaMKII to *I*_CaL_ and 1 *μ*M ISO, when *I*_Kr_ was blocked by 85%, EADs were induced, but when *I*_Kr_ blockage was reduced to 77%, EADs disappeared. These results are listed from row 6 to row 7 in Table 2.

## 4. Discussion

This study developed a modified computational model of human ventricular myocardium cell based on the ORd human model with the integration of regulation mechanism by CaMKII and PKA [20], with which their effects on EADs have been investigated.

EADs often occur during bradycardia under the condition of reduced repolarization reserve. O'Hara's group successfully elicited EADs with cycle length reduced to 4000 ms and *I*_Kr_ blocked by 85% [20]. In our simulation, with the cycle length halved (2000 ms) and *I*_Kr_ blocked by 85%, alternated EADs occurred with the sole effect of CaMKII overexpression to *I*_NaL_, suggesting that ventricular myocardial cell with CaMKII overexpression is more susceptible to EADs in normal HR range (CL = 2000 ms). Additionally, previous work has shown that *I*_CaL_ plays an important role in the occurrence of EADs [6]. Our simulation showed that CaMKII overexpression slightly increased *I*_CaL_ amplitude, but this effect alone did not induce EADs, and CaMKII did not alter the overlap region of *I*_CaL_ steady state activation and reactivation curves. Zaza et al. reported that [28], when repolarization is suitably slow, channel reactivation within the overlap region may break the current balance and support the possibility of autoregenerative depolarization. Other inward currents such as Na^+^-Ca^2+^ exchange current (*I*_NaCa_) may play certain roles in triggering EADs if amplitude is augmented and falls into the overlap region. Our simulation results suggest that CaMKII enhances these currents but does not induce EADs via this effect individually. Therefore, when CaMKII is overexpressed, the susceptibility to EADs is mainly originated from *I*_NaL_ variation.

Our study also demonstrated the behavior of ventricular myocardial cell with the integration of CaMKII overexpression and ISO application. With the ISO application, cyclic AMP (cAMP) is formed through *β*-adrenergic mediated activation of adenylyl cyclase, which activates PKA, a well-described mediator with targets that promote myocardial performance. In the case where PKA took effect independently, EADs occurred with shorter cycle length (2000 ms versus 4000 ms), indicating that the precondition of EADs is relaxed. Our results were different from that from Xie et al.'s study, where the APD shortening after ISO application was observed without EADs in steady state [19]. Xie et al.'s results could be caused by the simulation of transient *I*_CaL_ recovery and the prevented spontaneous SR Ca^2+^ release, limiting the *I*_NaCa_ in forward mode. Therefore, when the cells step into steady state with small inward *I*_NaCa_, the shortening of APD could be reasonable. In our work, SR Ca^2+^ release was enhanced by *I*_CaL_ amplification. With the integration with *I*_Kr_ blockage, *I*_NaCa_ was more likely to work as inward current, contributing to the prolongation of APD and occurrence of EADs. Additionally, the shortening of APD was also obtained without *I*_Kr_ blockage when *β*-adrenergic pathway was activated. Simulation results showed that when cycle length was chosen at 500 ms, 1000 ms, 1500 ms, 2000 ms, and 4000 ms, the corresponding APD90 shortening was 31.6 ms (233.0 versus 201.4 ms), 30.5 ms (268.6 versus 238.1 ms), 22.3 ms (282.6 versus 260.3 ms), 17.3 ms (289.1 versus 271.8 ms), and 18.6 ms (305.6 versus 287.0 ms). These results were consistent with the experiment measurements from Volders et al. [29]. In the case that CaMKII overexpression acted on *I*_NaL_ alone with ISO application, EADs occur at CL = 2000 ms and *I*_Kr_ blockage by 77%, suggesting that the combination of PKA and CaMKII overexpression on *I*_NaL_ relaxes the precondition further (*I*_Kr_ blockage 77% versus 85%) and increases the probability of EADs. With the CaMKII overexpressed on *I*_CaL_ alone, PKA induced EADs occurrence with 85%  *I*_Kr_ blockage, not 77%  *I*_Kr_ blockage, suggesting that, with the ISO application, *I*_NaL_ phosphorylation by CaMKII has more effect on EADs than *I*_CaL_. The steady state of CaMK_active_ was simulated with and without ISO application when CL = 2000 ms, and their corresponding maximum values were 0.0469 and 0.0421, respectively. This suggests that CaMKII activation increased with the application of ISO.

Our simulation results have shown that our proposed model is useful for exploring the interaction of *β*-adrenergic receptor signaling and CaMKII in formation of EADs; some potential limitations need to be addressed. Firstly, the role of CaMKII on RyR function has not been considered. There is growing evidence about its role in modulating RyR function. Increasing Ca^2+^ leak via RyR would limit SR Ca^2+^ load and disturb Ca^2+^ cycling. This should be incorporated into an improved model in a future study. Secondly, CaMKII overexpression may downregulate inward rectifier potassium current (*I*_K1_) and increase baseline *I*_K1_ amplitude [30]. We did not include any CaMKII effect on *I*_K1_ in our model. Thirdly, our model only describes the short-term effect of CaMKII on different current targets. It has been suggested that short-term and chronic effects of CaMKII are different [30–33]. Short-term (milliseconds to hours) CaMKII overexpression may slow *I*_to_ inactivation and accelerate recovery, but chronic overexpression may downregulate *I*_to,fast_ and upregulate *I*_to,slow_ [34]. Therefore, there is a scope to improve our model by taking chronic effects of CaMKII into consideration in future. Fourthly, there is a lack of an accurate measurement about how ISO regulates *I*_NaK_ with dynamic calcium change during the beat cycle, although it has been published by Gao et al. [35] that ISO regulated I_NaK_ increased (when calcium is 1.4 *μ*M) or decreased in pump current (when calcium is 0.15 *μ*M) depending on the intracellular Ca^2+^ concentrations. In our model the resting calcium concentration was 0.12 *μ*M and the peak calcium concentration was 1.1 *μ*M. Similar to the simulation work of Heijman et al. [26], by simply increasing the pump current from 17% to 33%, no significant change of *I*_NaK_ in triggering EADs (results not shown here) has been observed. However, when the continuous experimental data from human ventricular cells is available, the regulation of ISO on *I*_NaK_ could be incorporated into the model to reconfirm this nonsignificant effect. Fifthly, it has been reported that local regulation of cAMP and substrate phosphorylation play important roles in *β*-adrenergic receptor signaling [26, 36–38], so it could be useful to incorporate local control mechanism in a future study. Lastly, there are four CaMKII isoforms (*α*, *β*, *γ*, *δ*) with different distributions, kinetics, and roles in physiological and pathological adjustments. Until now, these differences have not been fully understood. Developing a model with detailed CaMKII isoforms information could be useful when experiment data are available.

In conclusion, our simulation results computationally demonstrated a better understanding of the combinational effect of CaMKII and ISO stimulus on the occurrence of EADs in human ventricular myocyte, which may provide useful tool to research therapeutic methods for the treatment of arrhythmia.

## Figures and Tables

**Table 1:** Current increment and EADs occurrence when different targets were phosphorylated by CaMKII independently.

Cycle length (ms)	*I*_Kr_ blockage level (%)	CaMKII target (CaMK_0_ = 0.12)	Target current variation	Deactivation time (ms)	EADs
2000	85	*I*_NaL_	Decreased by 24% (from 0.25 to 0.19 *µ*A/*µ*F)	1297 versus 761 ms	Alternated
		*I*_CaL_	Increased by 4.2% (from 1.67 to 1.74 *µ*A/*µ*F)		No
		*I*_CaK_	Increased by 6.5% (from 0.62 to 0.66 *µ*A/*µ*F)		No
		*I*_CaNa_	Increased by 8.1% (from 0.37 to 0.40 *µ*A/*µ*F)		No
		*I*_to_	Increased by 3.2% (from 0.95 to 0.98 *µ*A/*µ*F)		No

**Table 2:** Combined effect of CaMKII overexpression and *β*-adrenergic agonist on EADs.

Cycle length (ms)	ISO application	CaMKII target (CaMK_0_ = 0.12)	*I*_Kr_ blockage level (%)	EADs
2000	None	None	85	None
	1 *µ*M	None	85	Yes
		None	77	No
		*I*_NaL_	85	Yes
		*I*_NaL_	77	Yes
		*I*_CaL_	85	Yes
		*I*_CaL_	77	No
